# Selenium as diagnostic marker of cancers

**DOI:** 10.1186/1897-4287-13-S2-A3

**Published:** 2015-11-26

**Authors:** Katarzyna Jaworska-Bieniek, Magdalena Muszyńska, Katarzyna Kaczmarek, Wojciech Marciniak, Grzegorz Sukiennicki, Marcin Lener, Katarzyna Durda, Tomasz Gromowski, Tomasz Huzarski, Tomasz Byrski, Jacek Gronwald, Oleg Oszurek, Cezary Cybulski, Tadeusz Dębniak, Antoni Morawski, Anna Jakubowska, Jan Lubiński

**Affiliations:** 1 Department of Genetics and Pathology, International Hereditary Cancer Center, Pomeranian Medical University, Szczecin, Poland; 2 Read - Gene, S.A., Grzepnica, Poland

## Aim of the study

The aims of the study were:

1. To evaluate correlation between the level of Se and occurrence of lung, laryngeal, colorectal and prostate cancers (retrospective study)

2. To determine risk of breast cancer depending on Se level (prospective study)

## Material and methods

The study was performed in five groups. For four of them (cancers of the lung, larynx, colon, prostate) and matched healthy controls serum was collected before treatment but during diagnosis. Whereas for breast cancer group serum was collected 1 - 2 years before tumor diagnosis. Cases and controls in each study groups were matched for year of birth (+/-3 years), number and location of cancer among Iº relatives, smoking - the number of pack years (+/- 10%) and adnexectomy (only for breast cancer group).

Se level in serum was determined in all individuals by inductively coupled plasma mass spectrometry (ICP-MS) using Elan DRC-e ICP-Mass Spectrometer, Perkin Elmer. For selenium measurements strong quality control criteria was applied: reference material (SeronoformTM) was measured every four samples, cases and controls samples were tested alternately, correction value: +/- 5%.

After obtaining results from mass spectrometry, individuals in each group were divided into four categories (quartiles). Comparison of number of cases and controls was performed in each quartile. To evaluate the occurrence of cancers, individuals with the lowest selenium level (quartile) was taken as reference category.

## Results

It was observed that detailed analysis of selenium concentrations showed that in groups of lung, laryngeal, colorectal and prostate cancers, and low Se level in serum is associated with an increase of cancers frequency. It was also observed that when Se level is higher the number of cancers decreases. In contrast, selenium level >100 μg/l in serum was associated with increased incidence of breast cancer (OR 5.89; p = 0.0004; CI = 2.19 - 15.84).

## Conclusions

1. low level of selenium may be diagnostic marker for selection of people to:

- CT for lung cancer cases

- laryngeal examination for larynx cancer cases

- colonoscopy- colon cancer cases

- prostate examination (PSA, biopsy) - prostate cancer cases

2. Se level above 100 μg/l is a marker of high risk of breast cancer

